# P02-016 - A novel PSMB8 mutation causing candle syndrome

**DOI:** 10.1186/1546-0096-11-S1-A123

**Published:** 2013-11-08

**Authors:** V Messia, M Pardeo, R Nicolai, C Bracaglia, F De Benedetti, A Insalaco

**Affiliations:** 1 Division of Rheumatology, Department of Pediatric Medicine, Ospedale Pediatrico Bambino Gesù, Rome, Italy

## Introduction

Chronic atypical neutrophilic dermatosis with lipodystrophy and elevated temperature (CANDLE) syndrome is a newly described autoinflammatory disease, which had been recently reported in 9 patients. It is characterized by onset during the first year of life of recurrent fevers, purpuric skin lesions, arthralgia, progressive lipodystrophy, hypochromic or normocytic anemia, delayed physical development and increased levels of acute phase reactants.

## Case Report

A 10 year-old young girl presented at 10 months of age with recurrent fevers, hepato-splenomegaly and nodular erythematous skin lesions of trunk and limbs; subsequently she progressively developed lypodistrophy, arthralgia and arthritis and edema of eyelids. She started steroids and, then, cyclosporine with partial benefit and with recurrence of symptoms following tapering and/or discontinuation. Her weight and height were below the 5^th^ percentiles with partial growth hormone defect. Skin biopsy showed typical features of lobular panniculitis. Laboratory tests showed persistent elevated acute phase reactants and Serum amyloid A levels persistent chronic anemia, mild recurrent leucopenia (minimum neutrophil count 1040), thrombocytopenia (minimum 94.000) and decreased IgA, IgG and IgM levels.Immunological and cytogenetic studies performed on bone marrow were normal. Response to hydroxychlorochine or colchicine was unsatisfactory. Subsequently, the patient developed severe proteinuria. Renal biopsy revealed a minimal change glomerulopathy; she was started on a standard nephrotic syndrome high-dose steroid protocol with remission of proteinuria.Complete sequencing of TNFRSF1A and MVK genes showed no mutations. Molecular analysis of PSMB8 (proteasome subunit β type 8) gene revealed the presence of c.208A>T p.(Thr70Ser) variant in heterozygotic status that has never been reported before. Because of a persistent inflammatory state, she was started on daily therapy with Anakinra (2 mg/Kg/die), discontinued after 10 days for absence of response. She is currently managed with chronic low dose glucocorticoids.

## Discussion

The similarities in the clinical phenotype of this case with those described by Liu et al support the conclusion that this novel variant Thr70Ser in the PSMB8 gene is a causative mutation. Minimal change glomerulopathy has not been reported in CANDLE patients. It may be a casual association: however, one of the 9 original patients is described as having nephrotic syndrome. Our patient also did not respond to Anakinra. A better understanding of the pathophysiology of the disease is needed to improve its management.

## Disclosure of interest

None declared.
